# SmSak, the Second Polo-Like Kinase of the Helminth Parasite *Schistosoma mansoni:* Conserved and Unexpected Roles in Meiosis

**DOI:** 10.1371/journal.pone.0040045

**Published:** 2012-06-29

**Authors:** Thavy Long, Mathieu Vanderstraete, Katia Cailliau, Marion Morel, Arlette Lescuyer, Nadege Gouignard, Christoph G. Grevelding, Edith Browaeys, Colette Dissous

**Affiliations:** 1 Center for Infection and Immunity of Lille, Inserm U1019, CNRS-UMR 8204, University Lille Nord de France, Institut Pasteur de Lille, Lille, France; 2 EA 4479, IFR 147, Universite Lille 1 Sciences et Technologies, Villeneuve d’Ascq, France; 3 Institute for Parasitology, Justus-Liebig-University Giessen, Giessen, Germany; James Cook University, Australia

## Abstract

Polo-like kinases (Plks) are a family of conserved regulators of a variety of events throughout the cell cycle, expanded from one Plk in yeast to five Plks in mammals (Plk1-5). Plk1 is the best characterized member of the Plk family, homolog to the founding member Polo of *Drosophila*, and plays a major role in cell cycle progression by triggering G2/M transition. Plk4/Sak (for Snk (Serum-inducible kinase) akin kinase) is a unique member of the family, structurally distinct from other Plk members, with essential functions in centriole duplication. The genome of the trematode parasite *Schistosoma mansoni* contains only two Plk genes encoding SmPlk1 and SmSak. SmPlk1 has been shown already to be required for gametogenesis and parasite reproduction. In this work, *in situ* hybridization indicated that the structurally conserved Plk4 protein, SmSak, was largely expressed in schistosome female ovary and vitellarium. Expression of SmSak in *Xenopus* oocytes confirmed its Plk4 conserved function in centriole amplification. Moreover, analysis of the function of SmSak in meiosis progression of G2-blocked *Xenopus* oocytes indicated that, in contrast to SmPlk1, SmSak cannot induce G2/M transition in the absence of endogenous Plk1 (Plx1). Unexpectedly, meiosis progression was spontaneously observed in Plx1-depleted oocytes co-expressing SmSak and SmPlk1. Molecular interaction between SmSak and SmPlk1 was confirmed by co-immunoprecipitation of both proteins. These data indicate that Plk1 and Plk4 proteins have the potential to interact and cross-activate in cells, thus attributing for the first time a potential role of Plk4 proteins in meiosis/mitosis entry. This unexpected role of SmSak in meiosis could be relevant to further consider the function of this novel Plk in schistosome reproduction.

## Introduction

Polo-like kinases (Plks) are important regulators of the cell cycle progression during M-phase, primarily involved in the assembly and dynamics of the mitotic spindle apparatus and in the regulation of the activation of cyclin-dependent protein kinases (Cdks) [1], [2]. Plks are found in all eukaryotic lineages other than plants and apicomplexans and form a family of serine/threonine (S/T) kinases with conserved cellular functions. Metazoans possess multiple Plks which have different functions but a common structure composed of a conserved amino-terminal S/T kinase domain and a carboxy-terminal Polo-box domain (PBD) responsible for protein-protein interactions and subcellular localization [3]. Five Plks (Plk1-5) are present in vertebrates [4].

Plk1-2-3 have similar structures with two conserved polo boxes that can dimerise intramolecularly and form a pocket for binding to phospho S/T residues (inside of the S-pS/pT-P-X sequence) inducing conformational changes and kinase activation [5], [6]. Plk1 is the best characterized member of the Plk family. It is homologous to Polo, the first Plk member discovered in *Drosophila* [7]. Plk1 participates in multiple functions in mitosis, including centrosome maturation, kinetochore–spindle attachment, chromosome segregation and cytokinesis. Over-expressed during late G2 and M phases, Plk1 is a key inducer of mitosis and meiosis [3], [8], and it already constitutes a validated target for anti-cancer therapy [9], [10]. In contrast to Plk1, Plk2 is expressed primarily in G1 phase and may control S phase entry, and Plk3 which is expressed at a constant level throughout the cell cycle is involved in different stress-response pathways. The roles of Plk2 (also named Snk for Serum inducible kinase) and Plk3 (Fnk for FGF-inducible kinase) are not completely understood [4].

Plk4 (also named Sak for Snk/Plk-akin kinase) is a divergent and unique member of the Plk family [11]–[13]. Its PBD contains only one polo box, suggesting that regulation and substrate repertoire of Plk4 are different from those of other Plks [8], [14]. During the cell cycle, Plk4 expression increases from late G1 to S phase, peaking at G2/M. Plk4 has a well-established function in centriole duplication [15], [16], but the recent discovery that Plk4 is involved in regulating cytokinetic exit broadens the role of this kinase in centrosome maturation and mitotic progression [17]. Its turnover must be strictly controlled, and this is achieved by an autoregulatory mechanism implying its autophosphorylation in PEST sequence, a recognition peptide for degradation by the proteasome [18], [19].

More recently, a fifth Plk family member, Plk5, has been identified in vertebrates. Mouse Plk5 has been shown to be inducible by stress as well as DNA damage, and it is localized in the nucleolus [20]. In humans, Plk5 is kinase-deficient and seems to have no role in cell cycle progression but specific functions in neuron differentiation and activity in tumor suppression [21].

In invertebrates, like the insect *Drosophila* or the nematode *Caenorhabditis elegans*, Plk genes belonging to the Plk1 and Plk4 subfamilies have been identified but no other Plk genes have been found. In the genome of the human platyhelminth parasite *Schistosoma mansoni*, we have found two genes, which similarly to other invertebrates, are orthologs of *Plk1* and *Plk4*, the latter one was named *SmSak* [22]. SmPlk1 is a protein homologous to other Plk1 members with conserved functions in mitotic processes [23]. *SmPlk1* is abundantly transcribed in parasite gonads and the dramatic alteration of schistosome gonads caused by the kinase inhibitor specific for Plk1, BI2536 [9] suggested that SmPlk1 played a crucial role in gametogenesis and parasite reproduction [23].

This paper presents the structure of the *SmSak* gene and functional analyses of the SmSak protein. We analysed its expression in the different stages of the parasite and found a concentration of transcripts in female ovary and vitellarium. The expression of SmSak in the model of *Xenopus* oocytes confirmed its Plk4 conserved function in centriole amplification. Furthermore, comparative studies of the functions of SmSak and SmPlk1 in meiosis progression indicated that in contrast to SmPlk1 [23], SmSak could not trigger directly G2/M transition in G2-blocked *Xenopus* oocytes. Interestingly, we discovered that SmPlk1 and SmSak proteins, directly interact and crossactivate each other inducing kinase activity responsible for spontaneous meiosis resumption in G2-blocked oocytes. These data indicated for the first time that Plk1 and Plk4 proteins are able to cooperatively contribute to meiosis/mitosis entry, which attributes a novel role to Plk4 proteins.

## Results

### Molecular Cloning of SmSak

*S. mansoni* genome annotation [24] led to the identification of a sequence putatively encoding a protein kinase similar to Sak proteins (Genbank XP_002579066). In order to confirm the sequence, we cloned by RT-PCR amplification of adult worm RNA, the cDNA of SmSak (Genbank GU084154) from start to stop codons. The full-length sequence contains an open reading frame of 2712 bp coding for a protein of 903 residues with a calculated molecular mass of 101.6 kDa.

Screening of the *S. mansoni* genome database *(* www.schistodb.net *)* with the SmSak cDNA sequence indicated that *SmSak* was present as a single copy in the genome, found on the chromosome 1 (Smp_079410 identifier; Smp_scaffold 000292). The gene *SmSak* extends over 13 kb and is composed of ten exons of variable size ranging from 51 (exon 1) to 989 bp (exon 8). The size of introns varies from 144 pb to 3591 pb. In common with most of the schistosome genes characterized so far, all exon-intron junctions obeyed the GT-AG rule. Further extensive genomic analyses indicated that only two Plk genes were present in the *S. mansoni* genome, encoding SmPlk1 (described in [23]) and SmSak respectively.

### Sequence and Structure of SmSak

**Figure 1:**
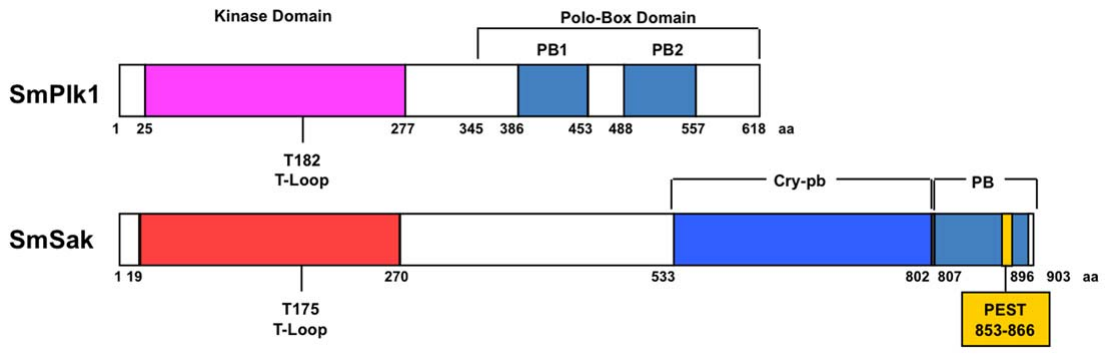
Structure of SmPlk1 and SmSak proteins. Plk1 and Sak proteins possess an amino-terminal serine/threonine kinase domain and a carboxy-terminal polo-box domain (PBD) that dictates the substrate specificity of the enzymes and regulates their function. Phosphorylation of a specific threonine (T_182_ or T_175_ respectively in SmPlk1 or SmSak) in the T-loop activation domain determines the activity of the kinases. Plk1 PBD is made up of two polo-boxes, PB1 and PB2, that form intramolecular heterodimers able to bind specific phospho-motifs. Binding to phosphosubstrates induces conformational changes and the access of the catalytic domain to its substrates [5], [6]. Both PB1(AA 386–453) and PB2 (AA 488–557) are contained in the sequence of SmPlk1. Sak PBD contains only one C-terminal PB, and a larger cryptic polo-box (cry-pb) weakly homologous to PB and unable to heterodimerize intramolecularly. However, PB from two Sak molecules can homodimerize and be involved in the regulation of the kinase activity. SmSak possesses a cry-pb (AA 533–802) and one PB (AA 807–896) containing a PEST destruction motif (AA 853–866) implicated very likely in the regulation of its turnover.

Protein sequence alignment displayed that SmSak and SmPlk1 share only 17% identity along their whole sequences and 37% identity in their kinase domains. Both proteins exhibit a conserved Plk structure composed of an N-terminal catalytic domain and a C-terminal PBD. The SmSak PBD is characteristic for Sak proteins and formed by a large cryptic polo-box (cry-pb) and a single polo-box (PB) domain instead of the tandem PB domains that are present in SmPlk1 (Figure 1) as well as in Plk1-3 of other species. Phylogenetic studies further confirmed that SmSak was a novel schistosome Plk belonging to the Sak/Plk4 group of the Plk family (Figure S1).

Blast analysis and sequence alignment of several Sak/Plk4 proteins (Figure S2) showed that the SmSak kinase domain (residues 19–270) contains the eleven subdomains characteristic of protein kinases, including SmPlk1 [23]. In subdomain I, the unique ATP-binding site GxGxFA sequence, common to other Plks, replaces the canonical GxGxxG motif present in the majority of protein kinases. The sequence HRDLxLxN (subdomain VI) and the typical motif GTPNYIxPE (subdomain VIII) which are considered as strong indicators for S/T kinase activity [25] are present in SmSak as well as in other Sak proteins. Moreover, the T_175_ residue located inside of the catalytic T loop of Plks and known to be the site of phosphorylation and kinase activation is conserved in SmSak, similarly to other Sak/Plk4 kinases. Overall, the kinase domain of SmSak showed greater identity to that of *Drosophila* Sak, human and murine Plk4 and Plx4 of *Xenopus* (55–57%) than to that of Polo kinases (SmPlk1, Polo, Plk1 and Cdc5) (27 to 37% identity).

SmSak_533–802_ and SmSak_807–896_ sequences correspond to the cry-pb and the PB of the protein, respectively (Figure 1). Both regions show a limited level of identity with the homologous domains found in other Sak/Plk4 proteins. The alignment of Sak proteins indicated that only 15–18% of the residues composing the cry-pb of SmSak are identical to other cry-pbs. The SmSak_807–896_ sequence showed greater identity to PB of human and murine Sak (25%) than to that of *Drosophila* (12%). Sak-PB domains have been shown to form homodimers, and the structure-based alignment of the PB sequences of diverse members of the Plk family has allowed the identification of 16 conserved residues positioned at the dimer interface [14]. Within the SmSak protein, 11 of these amino-acids are strictly conserved, suggesting that its PB domain can also dimerize (see Figure S2). Sak proteins are known to display a short half-life with PEST motifs governing their stability [11], [19]. Sequence analysis with ePESTfind (http://emboss.bioinformatics.nl/cgi-bin/emboss/epestfind) detected a single PEST sequence in SmSak located inside the PB at positions 853–866 (Figure 1). Whereas the PEST sequence commonly found behind the kinase domain of Sak proteins is not detectable in SmSak, the degron motif (ie, the starting place for degradation) DSGxxT occurring in this PEST sequence [19], seems to be conserved at this position in the SmSak sequence with the motif D_289_SIxxT (Figure S2). S_290_ and T_294_ residues are conserved. Their phosphorylation is known to create a binding site for β-TrCP (beta-transducin repeat containing), a component of the SCF (Skp1-cullin-F-box)-β-TrCP ubiquitin ligase complex, allowing the ubiquitination of Sak proteins [26], [27].

### Expression of SmSak in the Different Schistosome Stages

**Figure 2:**
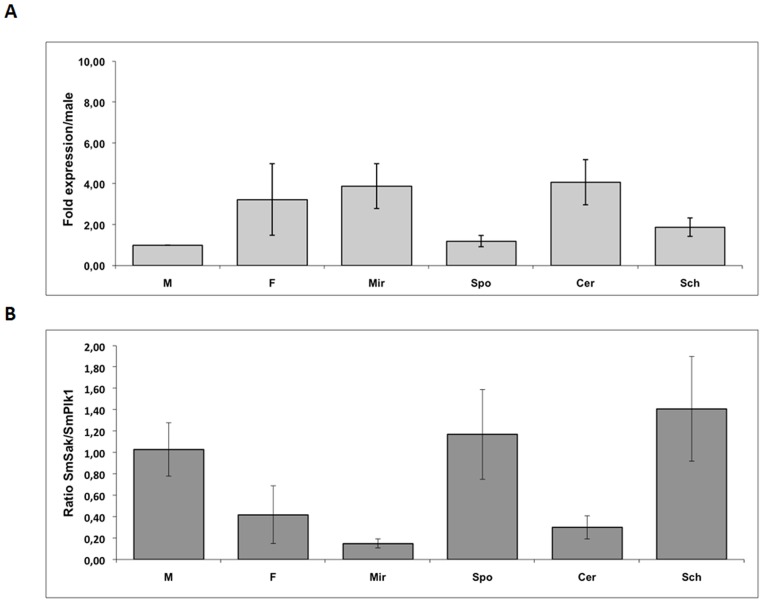
Quantification of SmSak transcripts in the parasite stages. *A. SmSak* transcripts were quantified in the different stages of the parasite *S. mansoni* (M, male; F, female; Mir, miracidia; Spo, sporocyst; Cer, cercaria; Sch, schistosomulum). Tubulin transcript level was used as internal standard. For graphical representation of qPCR data, ΔCt values calculated for *SmSak* in each parasitic stage were compared to the ΔCt value obtained for males using the deltadeltaCt (ΔΔCt) method [65]. Values were normalized as fold-difference relative to males. Mean of three determinations +/− SD. *B. SmSak/SmPlk1* ratios in each parasite stage. *SmSak* and *SmPlk1* transcripts were both quantified in two independent cDNA fractions prepared from each stage. In this case, SmPlk1 was used as a reference. Mean values of ratios +/− SD are given.

*SmSak* transcripts were quantified in the different life stages of the parasite by quantitative RT-PCR and transcript levels were compared to those of *SmPlk1* for each stage. As it was already shown for *SmPlk1* [23], the expression of *SmSak* appeared to be elevated in female worms and miracidia (Figure 2A). However, *SmSak* transcription dramatically decreases following transformation of miracidia into sporocysts, whereas here the *SmPlk1* transcript level remains to be stable [23]. *SmPlk1* and *SmSak* genes seem to be transcribed at similar levels in male worms whereas *SmSak* appears to be less transcribed than *SmPlk1* in female worms, cercariae and miracidia (66%, 70% and 85% less respectively) but more expressed than *SmPlk1* in sporocysts and schistosomula (17 and 41% more respectively) (Figure 2B).

**Figure 3:**
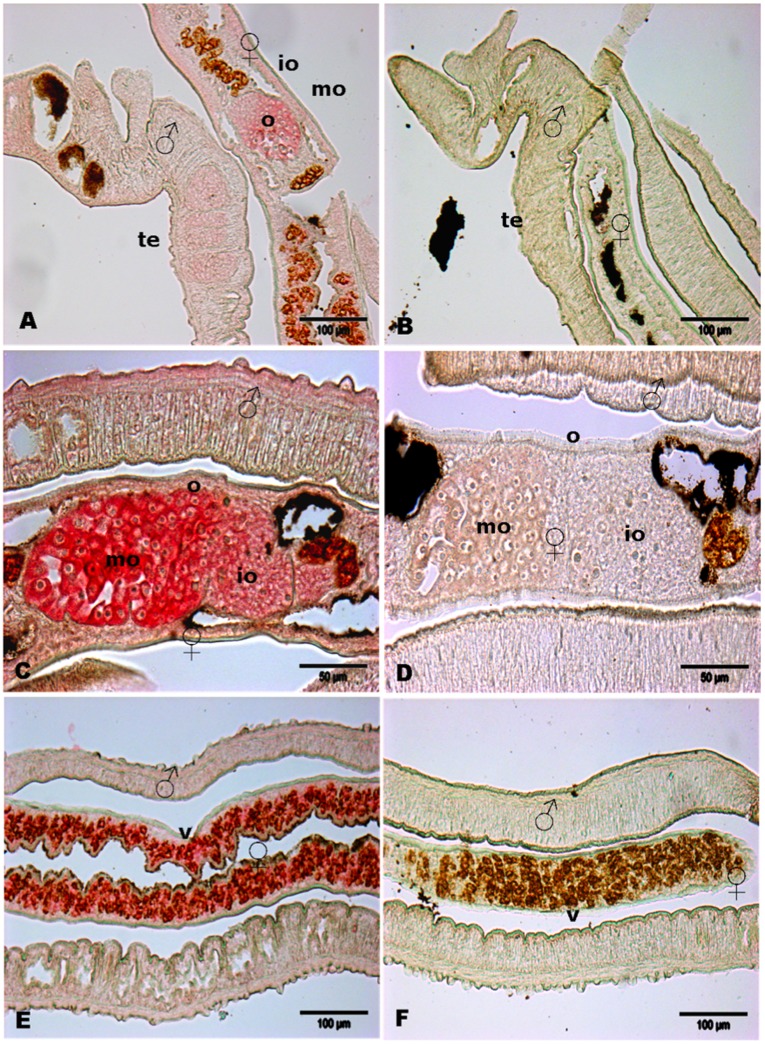
*In situ* localization of transcripts in adult worms. *In situ* hybridization experiments using DIG-labelled antisense- (A–C–E) and sense-RNA probes (B–D–F) of SmSak. *SmSak* transcripts were localized mainly in female reproductive organs: vitellarium and ovary in female worms (A–C–E) and to a lesser extent, in the testes of male worms (A). Scale bars = 100 µm (A–B–E–F), 50 µm (C, D). Abbreviations: te, testes; o, ovary; io, immature ovary; mo, mature ovary; v, vitellarium.

Detection of *SmSak* transcripts by *in situ* hybridization on adult worm sections indicated that SmSak is predominantly expressed in the ovary (mostly in the large ovocytes present in the mature part of the ovary) and in the vitellarium of females (Figure 3A,C,E). Although weakly, transcripts were also detected in the testicular lobes of the male (Figure 3A). The lower detection of *SmSak* transcripts in males than in females also correlates with the results obtained from Q-PCR (Figure 2A).

### Comparative Activities of *S. mansoni* Plks in *Xenopus* Oocyte Maturation

**Figure 4:**
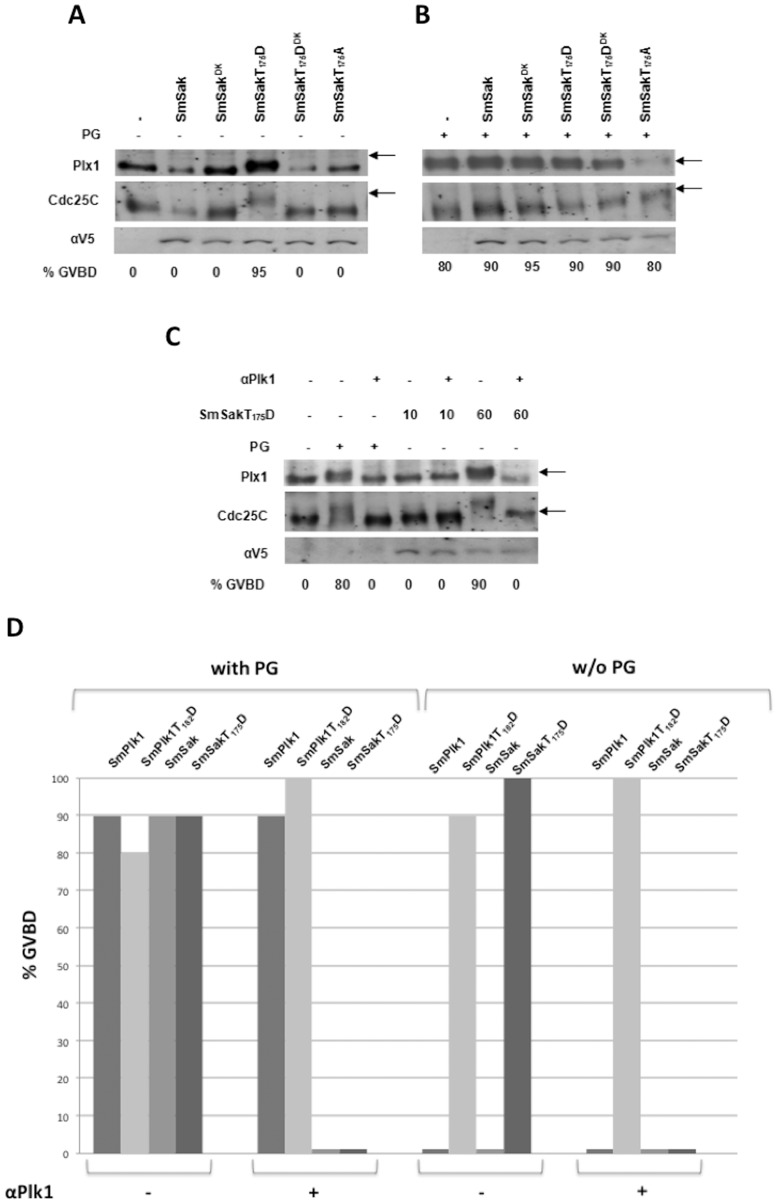
Analysis of SmSak activity in *Xenopus* oocytes. *A.* Constitutively active SmSak induces germinal vesicle breakdown (GVBD) and meiosis progression in *Xenopus* oocytes. Different samples of oocytes (n = 15) were microinjected with cRNA encoding SmSak, SmSak^DK^, SmSakT_175_D, SmSakT_175_D^DK^ or SmSakT_175_A. Percentages of oocytes exhibiting GVBD 18 h after microinjection of cRNA are indicated. Non-injected oocytes were used as control. Western blot analysis of oocyte extracts was performed as described in materials and methods to detect the gel-shift of endogenous phosphorylated Plx1 and Cdc25C proteins (indicated by arrows). The expression of the different forms of V5-tagged recombinant SmSak was confirmed by the use of anti-V5 antibodies. *B.* SmSak mutants do not alter the GVBD induced by progesterone. The experiment was performed as in *A*, except that PG was added to the different oocyte groups two hours after microinjection of cRNA preparations. Gel-shifts of Plx1 and Cdc25C are observed in all oocyte groups associated with high levels of GVBD. *C.* Endogenous Plx1 is required for the induction of GVBD by SmSakT_175_D. Oocytes were injected or not with 5 ng anti-Plx1 antibodies one hour before the injection of 10 or 60 ng SmSak_175_D cRNA. Meiosis resumption was monitored as in *A* and *B*. SmSakT_175_D induces GVBD (as already shown in *A*) following the injection of 60 ng cRNA but has no more effect in Plx1-depleted oocytes. In control oocytes, it was confirmed that anti-Plk1 antibodies blocked completely the GVBD induced by PG. *D.* Comparative analysis of the capacity of SmSak and SmPlk1 to induce GVBD in different conditions. Normal versus Plx1-depleted oocytes were incubated with or without (w/o) PG following the expression of SmPlk1, SmPlkT_182_D, SmSak or SmSakT_175_D. PG stimulates GVBD in the oocytes expressing each of the parasite kinases, provided that Plx1 is functional in oocytes. In Plx1-depleted oocytes, only SmPlk1 proteins are efficient. In the absence of PG, only constitutively active kinases are efficient, provided that Plx1 is functional. In Plx1-depleted oocytes, only SmPlkT_182_D can induce GVBD. These results were correlated with our previous data [23] and those presented in *A*, *B* and *C*. All percentages of GVBD represent the mean of three independent experiments. In *A*, *B* and *C*, blots are representative for the three experiments.

The main function of Plk1 kinases is to trigger G2/M transition in cells by activating Cdc25C phosphatase and by promoting the translocation of MPF (Maturation Phase Factor) into the nucleus. In *Xenopus*, evidence has been obtained that injection of cRNA encoding an active form of Plx1 (Plk1 homolog) was sufficient to initiate meiosis resumption in resting oocytes and to induce dissolution of the nuclear membrane or germinal vesicle breakdown (GVBD) [28], demonstrating the decisive role played by Plx1 in G2/M transition. Previous studies have shown that schistosome SmPlk1 was efficiently expressed in *Xenopus* oocytes and could trigger resumption of meiosis, bearing a function similar to that of Plx1. Indeed, in Plx1-depleted oocytes, the expression of SmPlk1 induced in the presence of the hormonal stimulus progesterone (PG), Cdc25 phosphorylation and MPF activation, leading to the entry in M phase and provoking germinal vesicle breakdown (GVBD) [23]. To unravel a possible function of SmSak, we have investigated its activity during *Xenopus* oocyte maturation. Results in Figure 4A indicate that the injection of cRNA encoding SmSak did not induce resumption of meiosis in oocytes, but that the expression of SmSakT_175_D, a mutant of SmSak in which the conserved residue T_175_ within the T-loop has been changed to aspartate in order to mimick its phosphorylation, induced spontaneously GVBD in unstimulated oocytes, with an efficiency comparable to that of PG stimulation (see Figure 4B). In these conditions, phosphorylations of Plx1 and of its substrate Cdc25C, were detected by protein shift in SDS-PAGE. These results confirmed that SmSakT_175_D is an active kinase and were in agreement with previous observations that the expression of non-regulated active kinases in oocytes consistently induced maturation [23], [29], [30]. When similar assays were performed in the presence of PG (Figure 4B), GVBD occurred in non-injected oocytes as well as in oocytes expressing SmSak or the active mutant SmSakT_175_D. The results also indicated that dead-kinase (SmSak^DK^) or unphosphorylable kinase (SmSakT_175_A) mutants of SmSak did not affect Plx1 and Cdc25 phosphorylations or oocyte maturation induced by PG, indicating that in contrast to SmPlk1^DK^ and SmPlk1T_182_V [23], inactive SmSak mutants did not represent negative competitors of Plx1. This result suggested that SmSak could not rescue the lack of Plx1, which was further confirmed by the demonstration that induction of GVBD by the constitutively active SmSakT_175_D required the presence of endogenous Plx1 in oocytes. Under the influence of injected anti-Plx1 antibodies, SmSakT_175_D was no more able to induce GVBD in oocytes. Additionally, we demonstrated that a minimal amount of SmSakT_175_D protein (expressed from 60 ng but not from 10 ng cRNA) was necessary to trigger mitotic entry (Figure 4C). A comparison of the capacity of SmPlk1 and SmSak (both native or constitutively active) to induce GVBD in normal versus Plx1-depleted oocytes with or without addition of PG is presented in Figure 4D. With PG, GVBD occurred in oocytes expressing either native or active SmPlk1 and SmSak, but if the oocytes were Plx1-depleted, maturation took place only in oocytes expressing SmPlk1 or SmPlk1T_182_D. In the absence of PG, active SmPlk1T_182_D and SmSakT_175_D induced GVBD in normal oocytes but only SmPlk1T_182_D could elicit maturation in Plx1-depleted oocytes. These data confirm those presented in Figure 4C and in [23] and allowed the conclusion that SmSak and SmPlk1 play distinct roles in cell cycle progression.

### Role of SmSak in *de novo* Centriole Formation

**Figure 5:**
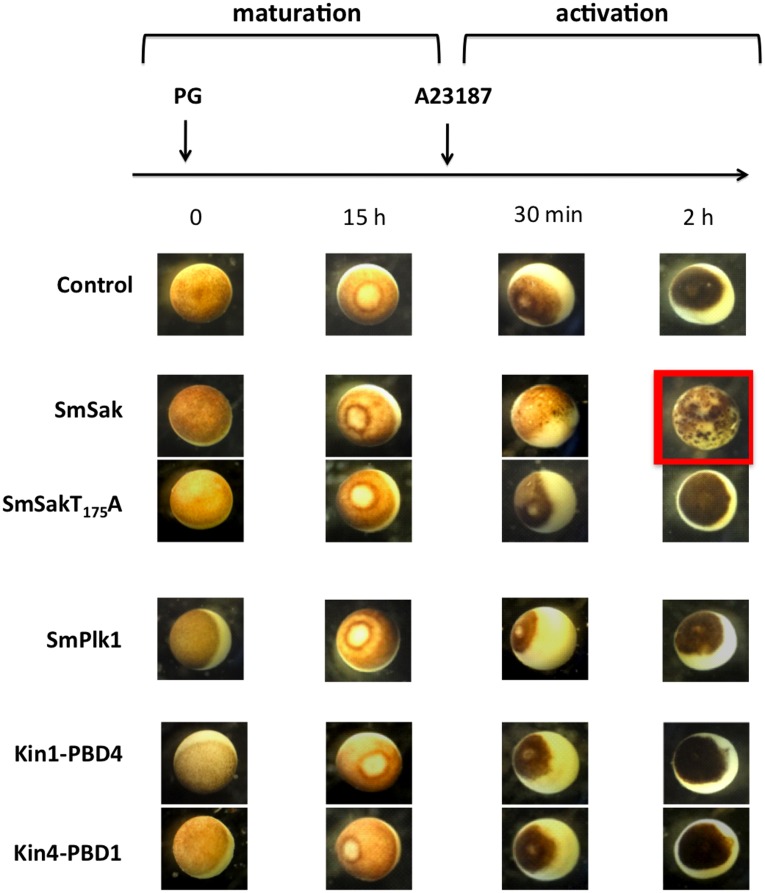
SmSak induces *de novo* centriole formation in *in vitro* matured *Xenopus* oocytes. Samples of cRNA encoding SmSak, SmSakT_175_A, SmPlk1 or Kin1-PBD4 and Kin4-PBD1 hybrid proteins, were microinjected in oocytes and incubated for 2 h for protein expression, before addition of PG. At 15 h following the addition of PG, GVBD monitored by the formation of a white spot centered at the germinal pole of Metaphase II arrested oocytes, was observed in all oocyte groups. Matured oocytes were then activated with calcium ionophore A23187, and surface morphology was observed after 30 min or 2 h. Only SmSak-expressing oocytes formed pigmented speckles that increased in number from 30 min to 2 h. In all other oocytes, the animal pole gradually darkened after the addition of A23187 and the formation of a black spot (fertilization coat) could be seen.

Plk4 is known to regulate centriole biogenesis [16], and its deregulation has been linked to tumor development [31]. Overexpression of Plk4 in human cells induced rapid formation of multiple complete centrioles within a single S phase [32]. Moreover, Sak overexpression was reported to induce amplification of centrioles in *Drosophila* embryos as well as their *de novo* formation in unfertilized *Drosophila* eggs [33], [34]. This process of *de novo* centriole formation due to Plk4 overexpression has been also demonstrated in vertebrates. Indeed, overexpression of Plx4, the *Xenopus* homolog of human Plk4, was shown to induce *de novo* centriole formation in activated *Xenopus* eggs, but not in immature (stage VI) oocytes [35]. Activation of eggs was artificially induced *in vitro* by the addition of the calcium ionophore A23187 to matured oocytes. A23187 mimics the increase of intracellular calcium level, which occurs soon after fertilization playing a crucial role for sperm-induced egg activation in almost all species [36]. According to this, we decided to study the potential function of SmSak on centriole duplication in matured *Xenopus* oocytes (Figure 5). Following a protocol similar to that described before [35], immature oocytes were injected with cRNA encoding SmSak constructs and incubated with PG in order to induce their maturation. Matured oocytes were then activated with calcium ionophore A23187 and the surface morphology of oocytes was monitored microscopically. Figure 5 shows that the expression of SmSak did not elicit any phenotype in PG-stimulated oocytes which underwent GVBD similarly to control oocytes and produced eggs blocked at metaphase II of meiosis. However, when these eggs were further activated with A23187, all the oocytes expressing SmSak developed speckled-like pigmentation on their surface within 2 h, whereas the surface of control (non-injected) oocytes remained unchanged. Pigmented speckles did not appear even after 2 h on the surface of eggs previously injected with SmSakT_175_A, indicating that the kinase activity of SmSak was essential to induce the speckled phenotype. Moreover, the results indicated that SmPlk1 expressing oocytes did not show pigmentation under activation by A23187, demonstrating the specific function of SmSak in this process (Figure 5).

### Roles of Kinase and PBD Domains of Schistosome Plks in Oocyte Maturation and Activation

**Figure 6:**
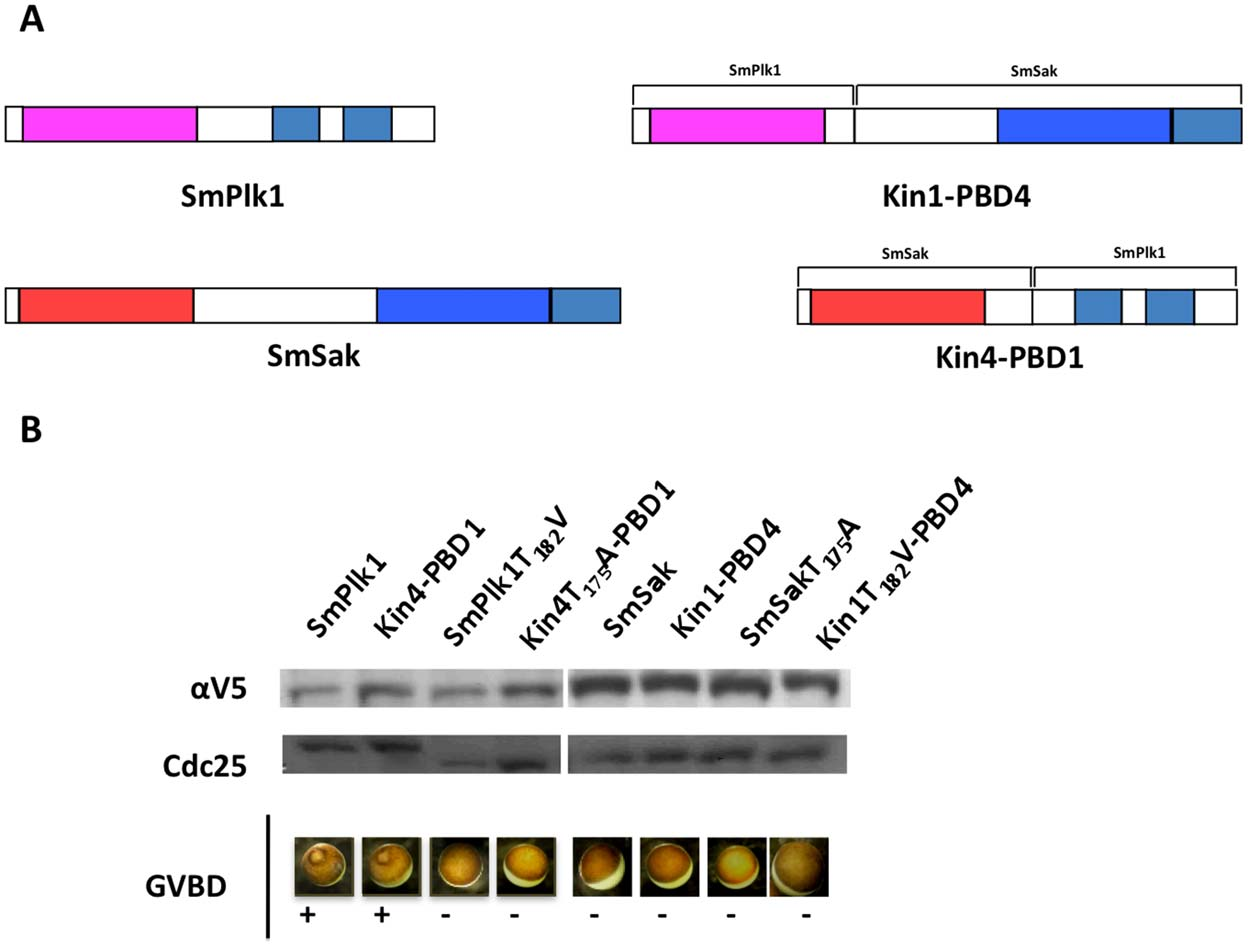
Studies of the importance of SmPlk1 and SmSak domains for oocyte maturation by using SmPlk1/SmSak hybrid constructs. *A.* Schematic representation of Kin1-PBD4 and Kin4-PBD1 hybrids formed by the fusion of the N-terminal part of SmPlk1 (AA 1–342) with the C-terminal SmSak (AA 323–618) and that of N-terminal SmSak (AA 1–322) with C-terminal SmPlk1 (AA 343–903), respectively (refer to Figure 1). *B.* Different sets of Plx1-depleted oocytes were microinjected with cRNA encoding SmPlk1, SmSak, Kin1-PBD4 or Kin4-PBD1, or their unphosphorylable variants, and stimulated by the addition of PG. GVBD monitored 18 h after microinjection of cRNA, only occurred in oocytes expressing SmPlk1 and Kin4-PBD1 that contain phosphorylable T_182_ and T_175_, respectively. Western blot analysis of oocyte extracts with anti-V5 antibodies confirmed the expression of proteins in oocytes. Gel shift of Cdc25 was detected only in matured oocytes.

To decipher the relative importance of kinase and PBD structures of SmPlk1 and SmSak for their respective functions, we have constructed two hybrid molecules composed of the kinase domain of SmPlk1 with the PBD of SmSak (namely Kin1-PBD4) and inversely of the kinase domain of SmSak with the PBD of SmPlk1 (namely Kin4-PBD1) (Figures 1 and 6A) and we have analyzed their activity in the process of oocyte activation described previously. First, we observed that neither Kin1-PBD4, nor Kin4-PBD1 were capable of generating the speckled phenotype obtained in SmSak-expressing matured oocytes when they were activated by A23187. This confirms the importance and the specificity of both the kinase and the PBD domains of Sak for centriole biogenesis [35] (Figure 5).

In contrast to SmPlk1, SmSak cannot restore the function of Plx1 in Plx1-depleted oocytes (Figure 4D). However, the results presented in Figure 6 clearly indicated that Kin4-PBD1 could rescue, as SmPlk1 (Kin1-PBD1), the absence of Plx1 in oocytes which underwent GVBD under PG stimulation, whereas this was not observed for Kin1-PBD4 or SmSak (Kin4-PBD4). These data confirmed that PBD1 was required for a Plk-dependent maturation of oocytes. Furthermore, the use of constructs in which Kin1 and Kin4 originated from unphosphorylable SmPlk1T_182_V and SmSakT_175_A demonstrated that the effect of Kin1-PBD1 and Kin4-PBD1 in oocyte maturation was also dependent on their kinase activities. Finally, these data showed that PBD1 was specifically required for the activity of Plks in oocyte maturation but they also indicated that both the SmPlk1 and the SmSak kinase domains could be functional in this process.

### Co-expression of SmPlk1 and SmSak is Sufficient to Induce Oocyte Maturation

**Figure 7:**
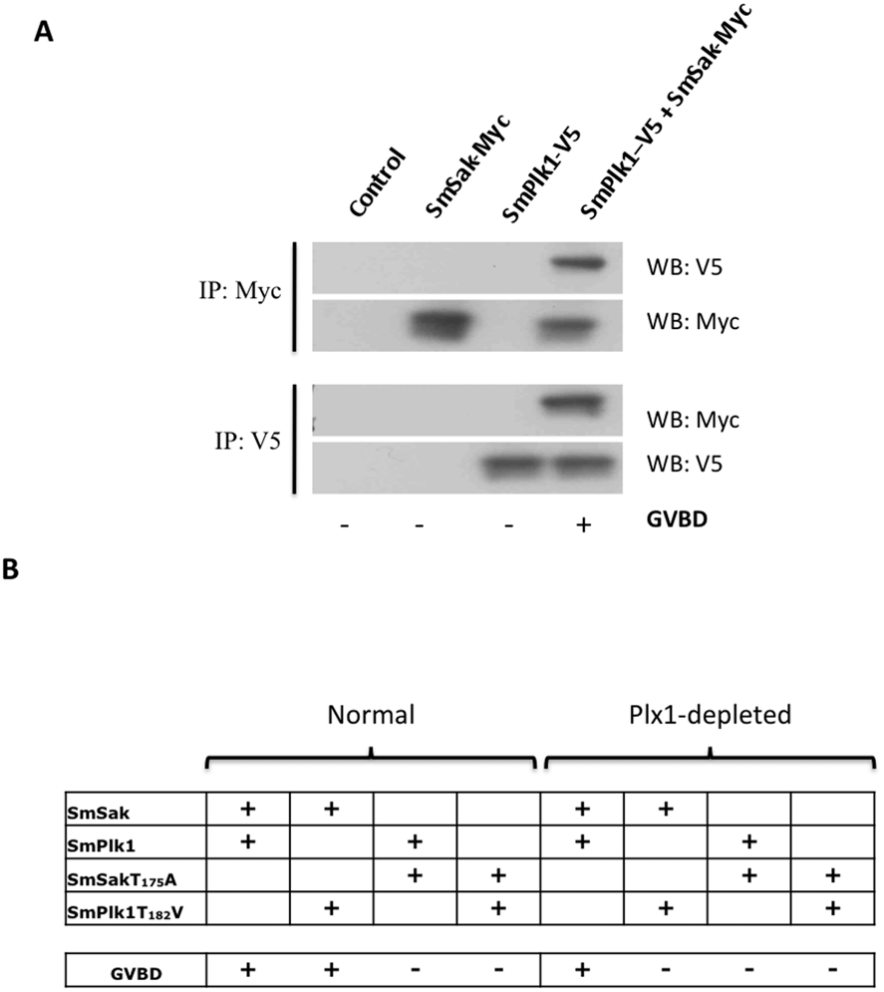
Interaction between SmPlk1 and SmSak and kinase co-activation. *A.* Co-immunoprecipitation of SmPlk1 and SmSak expressed in *Xenopus* oocytes. Oocytes were injected with V5 tagged-SmPlk1, Myc tagged-SmSak or with both kinases simultaneously. Non-injected oocytes were used as controls. Following 18 h, GVBD was monitored. Oocyte lysates were prepared and parasite kinases were immunoprecipitated with anti-V5 or anti-Myc antibodies, then analyzed by western blotting. Results show that immunoprecipitates contained both SmPlk1 and SmSak when the proteins were co-expressed. *B.* Co-activation of SmPlk1 and SmSak induces GVBD. In normal or Plx1-depleted oocytes, SmSak was expressed with SmPlk1 or with SmPlk1T_182_V and reciproqually SmPlk1 was expressed with SmSak or with SmSak T_175_A. SmPlk1T_182_V and SmSakT_175_A were also expressed together. Only the co-expression of SmSak and SmPlk1 induced GVBD in normal as well as in Plx1-depleted oocytes. All the other combinations were inefficient, except the co-expression of SmSak with SmPlk1T_182_V, that allowed GVBD but only in normal oocytes (containing a functional Plx1).

Results of *in situ* hybridization revealed a concentration of both SmPlk1 and SmSak transcripts in the vitellarium as well as in the oocytes present in the ovary of female schistosomes ([23] and Figure 3). This finding prompted us to investigate the possibility that SmPlk1 and SmSak might cooperate to regulate mitosis or meiosis in parasite germinal cells. We have previously shown that the expression of native SmSak (Figure 4 A, D) or SmPlk1 ([23], Figure 4D) in immature *Xenopus* oocytes was not sufficient to release their arrest in late G2 of meiosis I, but that these oocytes were capable of responding to a PG stimulus and to undergo GVBD, exactly as control oocytes. In this work, we have analyzed the consequences of the simultaneous expression of SmSak and SmPlk1 in such immature oocytes and discovered that GVBD occurred in these conditions without addition of PG. This suggests that an activation of at least one of the kinases had occurred, which we assume to result from an interaction between both parasite proteins. Co-immunoprecipitation experiments finally confirmed the association between SmSak and SmPlk1 proteins in oocytes which were injected with Myc-SmSak and V5-SmPlk1 constructs. Western blot analysis of proteins immunoprecipitated from oocyte lysates using anti-Myc or anti-V5 antibodies (Figure 7A) confirmed the correct expression of both proteins and showed that SmPlk1 was present within the anti-Myc immunoprecipitates and, inversely, that SmSak was present in anti-V5 complexes. In order to determine which kinase(s) was (were) responsible for GVBD, we tested unphosphorylable SmSakT_175_A and SmPlk1T_182_V in co-expression experiments. Results showed that the combination SmSakT_175_A/SmPlk1 was ineffective, whereas GVBD occurred with the tandem SmSak/SmPlk1T_182_V (Figure 7B). This indicated in this case that the kinase activity of SmSak was required, but not that of SmPlk1, for which deficient activity was probably rescued by that of endogenous Plx1. This hypothesis could be finally verified by the finding that maturation did not happen using SmSak/SmPlk1T_182_V constructs injected into Plx1-depleted oocytes. Additional experiments in which the hybrid constructs of SmPlk1 and SmSak (described in Figure 6A) were used, indicated that the co-expression of Kin1-PBD4 and Kin4-PBD1 led to GVBD and they also confirmed that both PBD1 and PBD4 were required for the activation process (results not shown).

## Discussion

*Schistosoma* is one of the most widespread human parasites, with several hundred million people infected in many tropical and subtropical countries [37], [38]. The severity of schistosomiasis is primarily due to the fecundity of the worms which develop in human hosts. Mating and sexual maturation of adult male and female *S. mansoni* in the host lead to the production of huge numbers of fertilized eggs, which are for a large part released with the feces, thus assuring active transmission. Besides, many eggs get trapped in host tissues which are responsible for the formation of inflammatory granulomas and for the pathology of schistosomiasis. Chemotherapy still remains the most efficient approach to control morbidity and mortality due to schistosomiasis and praziquantel (PZQ) has been the drug of choice for several decades. However prolonged and intensive use of PZQ in monotherapy has already raised serious concerns about the development of resistance and the necessity to discover novel targets and new drugs against schistosomes [39], [40]. Strategies for a reduction of parasite fertility would constitute valuable approaches towards the control of both transmission and pathology of the disease and in this context, recent studies have been undertaken to decipher the molecular mechanisms that regulate the development of sexual organs and gamete production in *S.mansoni* [41]–[44]. Following the clear demonstration that receptor tyrosine kinase signalling plays essential functions in schistosome reproduction processes [43], [44], and that parasite-specific tyrosine kinases could already represent novel interesting drug targets [29], [30], [44]–[45], we have further considered the importance of the mitotic kinases, Plks, in the regulation of cell cycle progression during schistosome reproduction processes. *S mansoni* is the only trematode in which Plks have been studied so far. Recently, we have characterized the first *Schistosoma* Plk, SmPlk1, and shown that its function is essential for parasite reproduction. BI2536 (the first-in-class prototype Plk1 inhibitor) inhibited SmPlk1 kinase activity and induced *in vitro* dramatic alterations in schistosome gonads, which affected oogenesis and spermatogenesis, suggesting that SmPlk1 could represent a new target against schistosomiasis [23].

Plk1 (Polo in *D. melanogaster*) [7] is the founding member of the Plk family that comprises five different members (Plk1-5) in mammals [4]. Plks are crucial mitotic regulators found in organisms as diverse as yeast and human. The expansion of the Plk family from one Plk gene in single cell eukaryotes (cdc5 and Plo1 in yeasts) [46], [47] to five genes in mammals (in mouse and human) [4], resulted in Plk diversification and likely enabled the increased complexicity of cell division in animals. Plk4 (also named Sak for Snk akin kinase) is a unique member of the Plk family, found in all animals, and in a few other lineages (fungi and ciliates) [4]. Plk4 has been considered as an odd-one out of the Plk family [19] since its PBD is formed by one cryptic-polo box and one PB being quite different from the PBDs of Plk1 and other Plks that are all formed by a PB tandem. As illustrated in Figure S1, Plk4 originated very likely from a Plk1-like ancestor. Indeed, results of phylogenetic analyses clearly distinguished the Plk4/Sak subfamily, inside of the Plk family, from the major group composed of Polo/Plk1 members and Plk2, Plk3 and Plk5 proteins.

Knock-out mice experiments have shown that only Plk1 and Plk4 were essential for embryonic growth and viability [4]. In the platyhelminth *S. mansoni*, only two genes have been found that encode these two Plks, the previously studied SmPlk1 [23] and the Plk4 homolog, SmSak. Both schistosome proteins are structurally similar to their orthologs in other species. No gene coding for Plk2, Plk3 or Plk5 was detected in the genome of *S. mansoni* [22], and the presence of only two Plks in schistosomes corresponds to the occurrence of only Plk1 and Plk4 homologs in other invertebrates like the fly *D. melanogaster* and the nematode *C. elegans* [4]. However, noticeably, in *C. elegans*, three different Plk1 proteins (Plc1, Plc2 and Plc3) are present [48], and the Plk4 homolog is an atypical Plk4/ZYG-1 protein functionally related to Plk4, but structurally distinct [49].

Gene exon-intron structure of *SmSak* (10 exons) was found to be more complex than that of *SmPlk1* (7 exons) [23], but less complex than that of the murine *Sak* gene that is formed by 15 exons [50]. It encodes the SmSak protein which has the conserved structure of Plk4 proteins but also their functional characteristics. SmSak functions were unravelled by its heterologous expression in *Xenopus* oocyte, one of the most appropriate cellular model to express schistosome proteins [45], [51], [52] and, furthermore, to study the function of molecules regulating mitosis and meiosis [23], [28].

SmSak contains a typical Plk kinase domain, characterized by the unique ATP-binding site GxGxFA sequence present in all Plks [53]. The essential threonine residue located in the catalytic T loop of Plks and responsible for their activation [8] is conserved in SmSak (T_175_),and we have shown that mimicking the phosphorylation of this threonine (by an aspartate mutation) is sufficient to induce constitutive activation of SmSak, as it was already described for SmPlk1 [23]. In *Xenopus* oocytes, the expression of SmSakT_175_D induced oocyte maturation, and this activity was insensitive to the Plk1-specific inhibitor BI2536 up to 200 nM (results not shown), while a dose of 20 nM already inhibited 100% of the activity of SmPlk1T_182_D in similar conditions [23]. These data were in agreement with the important structural differences observed between the kinase domains of SmSak and SmPlk1 (only 37% of identical residues) and confirmed that, in our previous experiments, gonad alterations in parasites treated *in vitro* with BI2536 were effectively the consequences of the specific targeting of SmPlk1 [23].

In contrast to the Plk4/ZYG1 protein of *C. elegans* [50], SmSak contains a conserved Plk4-like PBD formed by one cry-pb and one PB. Whereas these boxes present a relatively low level of identity with those of other Plk4/Sak proteins (see Figure S2), we have been able to show that they can mediate SmSak activation and its conserved functions in centrosome targeting and centriole duplication.

PB domains are structural motifs located at the C-terminus of any Plk. Tandem PB of Plk1-3 proteins can bind to specific phosphorylated proteins, forming intramolecular heterodimers that regulate the kinase activity by induction of conformational changes [8]. In Plk4 proteins, the single PB has been shown to form homodimers both *in vitro* and *in vivo* and this intermolecular homodimerization is involved in the regulation of kinase activity and trans-autophosphorylation [14]. Structure-based alignments of various PB sequences has shown that the PB hydrophobic core was composed of conserved residues, a large proportion of them being involved in PB dimer formation [14]. In the SmSak protein, many of these residue positions are conserved at the dimer interface (see Figure S2), suggesting that SmSak could also dimerize to self-activate and to auto-phosphorylate. Indeed, Plk4 autophosphorylation is an essential mechanism by which Plk4 levels are regulated along the cell cycle [18]. Plk4 proteins are short-lived proteins and their turn-over is achieved by an autoregulatory process in which the kinase domain autophosphorylates PEST motifs along the molecule, thus allowing its ubiquitinylation and its targeting to the proteasome for degradation [19]. A PEST sequence has been found in SmSak located inside of the PB. Also, the degron motif DSGxxT demonstrated to be essential for the regulation of Plk4 stability in many species [19] is relatively well conserved in SmSak and this lets suppose that SmSak turn-over could be also controlled, as other Plk4 proteins, by β-TrCP–mediated degradation [26], [27], [54].

Centrioles organize the formation of microtubule-derived structures, including cilia and centrosomes [55]. Centriole duplication needs to be tightly regulated and coordinated with the cell cycle, because abnormalities in centrosome number and structure cause genomic instability and cancer [56]. Different studies have shown that Plk4 is a key regulator of centriole duplication and required for an accurate reproduction of centrosomes during the cell cycle [15]–[18], [57]. Plk4 is an upstream regulator of canonical biogenesis necessary for centriole formation and its overexpression could induce amplification of centrioles in *Drosophila* embryos but also their *de novo* formation in unfertilized *Drosophila* eggs [34]. More recently, *de novo* centriole formation was induced in *in vitro*-matured oocytes of *Xenopus* following overexpression of Plx4 [35]. In this work, we have analyzed the effects of the expression of the heterologous SmSak protein in such oocytes and demonstrated that the parasite protein could exert the same activities as Plx4 for *de novo* centriole formation. Expression of a dead-kinase SmSak variant or of hybrid schistosome Plks (Kin1-PBD4 or Kin4-PBD1) did not generate the multicentriolar structures observed in oocytes expressing native SmSak. This indicated that both the kinase activity and the PBD interaction domains were required for the capacity of SmSak to induce centriole formation, which corresponds to previous results obtained for Plx4 [35]. A similar phenotype observed in *Xenopus* oocytes overexpressing Plx4 or the invertebrate SmSak confirmed the functional conservation of SmSak in centriole biogenesis.

Overall, our studies provide clear evidence that SmSak and SmPlk1 play distinct roles in cell cycle progression. We have demonstrated that SmSak, but not SmPlk1, plays a major role in centriole duplication while SmPlk1, but not SmSak, can trigger G2/M transition in *Xenopus* oocytes (Figure 4). These cells are arrested in G2 of meiosis 1 because they contain inactive MPF complexes that need to be dephosphorylated by the phosphatase Cdc25 to allow the entry in M phase. Plx1 (as all Plk1 proteins including SmPlk1) is a major activator of Cdc25 and therefore a key inducer of mitosis [28]. The action of Plx1 is simultaneously dependent on its kinase activity and on its specific PBD that assures its correct subcellular localization and substrate interactions [28]. We have clearly confirmed that SmSak, in contrast to SmPlk1, was not able to induce GVBD in Plx1-depleted oocytes. Interestingly, we have found that the hybrid kinase Kin4-PBD1 could perfectly exert the functions of SmPlk1. One explanation is that the hybrid Kin4-PBD1, which could be targeted correctly to Plk1 substrates via PBD1, contained a Kin4 domain able, as Kin1 of SmPlk1, to phosphorylate *Xenopus* Cdc25. This hypothesis is in agreement with published data showing that human Plk4 can phosphorylate and exert an additional control on Cdc25C [58]. This information, together with the demonstration that *SmSak* transcripts, as those of *SmPlk1* [23], were abundant in the large oocytes of the female ovary (ie, in cells that could tentatively be compared with stage VI *Xenopus* oocytes arrested at G2 of meiosis 1) (Figure 3) prompted us to reinvestigate the potential participation of SmSak in meiosis resumption, especially via its possible cooperation with SmPlk1. Surprisingly, we could demonstrate in this work that the simultaneous expression of SmSak and SmPlk1 in immature *Xenopus* oocytes induced spontaneously GVBD, suggesting that both kinases could interact and self or cross-activate. An interaction between SmPlk1 and SmSak was confirmed by co-immunoprecipitation of both kinases, demonstrating for the first time that Plk1 and Plk4 proteins have the capacity to interact, and that this interaction could regulate their activity in cell cycle progression. Very likely, this interaction is mediated by the respective PBD of SmPlk1 and SmSak since induction of GVBD also occurred in the oocytes expressing hybrid constructs (Kin1-PBD4 and Kin4-PBD1). Further investigations are needed to determine the specificity of this novel interaction between SmPlk1 and SmSak proteins in detail. Our results strongly suggest that both SmPlk1 and SmSak kinases should be active in this process since the expression in Plx1-depleted *Xenopus* oocytes of unphosphorylable and thus non-activable SmSak or SmPlk1 had no more effect on meiosis resumption. Although no precise scheme can be drawn at this time for the mechanism of kinase activation, this observation could be of relevance to understand the regulation of meiosis in schistosomes, and strongly suggested the importance of SmSak, besides SmPlk1 which has been shown to co-localize with SmSak in reproductive organs [22], [23], in gametogenesis and parasite reproduction. It would be interesting to know whether a similar cross-talk between Plk1 and Plk4 proteins could turn out to be a more general phenomenon, occurring in other animal species. To a larger extent, we could also conceive that such an activating interaction between these two major mitotic kinases would have also crucial consequences in mitosis and cancer for cells in which their expression would be desynchronized.

In conclusion, we have shown that the second Plk of *S. mansoni*, SmSak, possesses the conserved structural and functional properties of Plk4 proteins and could play a major role in parasite gametogenesis. The presence of large amounts of *SmSak* transcripts in female ovary and vitellarium corroborates previous data in vertebrates, showing the expression of Plk4 in tissues containing actively dividing somatic and germ cells. However, a discrepancy appears between the localization of Plk4 in schistosome and vertebrate tissues. Indeed, SmSak is more expressed in female than in male reproductive organs whereas mouse Sak [11] and human Plk4 [13] were detected at the highest level in testes but not in ovaries, and that mutations in Plk4 caused male hypogonadism and germ cell loss in mice, suggesting a role for Plk4 in the initiation of spermatogenesis [59].

Further work is needed to understand precisely the role of SmSak in gametogenesis but SmSak already appears as a novel interesting target to combat schistosomiasis, with regard to its unexpected roles described here in meiosis. An attractive goal of this study was to confirm a role of SmSak in the development of reproductive organs and gametogenesis by knocking-down SmSak by RNA interference (RNAi), an approach already applied with success for schistosome kinases [29]. However, our different attempts to knock-down SmSak by RNAi have been unsuccessful, suggesting that SmSak belongs to the group of non-knockable genes [60]. Also, at this time, no drug inhibiting specifically the kinase activity of Plk4 proteins is available. It would be interesting to pursue the characterization of the kinase domain of SmSak in order to design specific inhibitors for SmSak and analyze their effects on parasite fecundity.

## Materials and Methods

### Ethics Statement

All experiments involving hamsters within this study have been performed in accordance with the European Convention for the Protection of Vertebrate Animals used for Experimental and other Scientific Purposes (ETS No 123; revised Appendix A) and have been approved by the committee for ethics in animal experimentation of the region Nord Pas de Calais France (authorisation No. AF/2009) in the local animal house of the Pasteur Institute of Lille (Agreement No.A59-35009).

### Parasite Material

A Puerto-Rican strain of *S. mansoni* was maintained by passage through albino *Biomphalaria glabrata* snails and *Mesocricetus auratus* hamsters. Cercariae and schistosomula were prepared as previously described [61]. Sporocysts were obtained from miracidia transformed *in vitro* by incubation in minimum salt medium at 28°C for 18 h [62]. Adult schistosome pairs were collected by portal perfusion from infected hamsters at 42–45 days p.i. Males were separated from females with a fine brush.

### Cloning of SmSak cDNA

Total RNA was isolated from adult schistosome pairs and purified by centrifugation through a caesium chloride gradient [63]. cDNA was prepared using the reverse transcriptase Thermoscript™ (Invitrogen). The SmSak sequence was amplified by PCR using as primers SmSakfwd (5′-GCAGATATCATGGATGTGGATTATCC-3′) and SmSakrev (5′-GAGCGGCCGCAGAAATAATTTTGTTGGC-3′) containing respectively *EcoRV* and *NotI* restriction sites and subcloned into the pCR2.1 TOPO cloning vector (Invitrogen). The full-length sequence of *SmSak* was confirmed by sequencing (GATC Biotech).

### Sequence Analyses

The genomic database of *S. mansoni* [24] was screened using the blastn algorithm (http://blast.ncbi.nlm.nih.gov.gate2.inist.fr/Blast.cgi) with the SmSak cDNA sequence (GenBank accession N°GU084154) as a probe, allowing us to determine by eye 5′-GT and 3′-AG intron-exon junctions.

Protein sequence analysis of SmSak and alignment with other Plks were performed using programs included in the Lasergene package (DNASTAR Inc., Madison, WI, USA). SmSak PEST motif was found using the ePESTfind server (http://emboss.bioinformatics.nl/cgi-bin/emboss/epestfind). For phylogenetic analyses, protein sequences of diverse polo-like kinases were aligned using ClustalW algorithm (BioEdit). Neighbour joining tree was then generated with the program MEGA5, using Dayhoff model and 1000 bootstrap repetitions.

### SmSak Plasmid Constructs

*SmSak* cDNA was cut using *EcoRV* and *NotI* restriction sites from pCR2.1 and inserted in-frame into a V5/His-tagged pcDNA3.1 expression vector (Invitrogen). The SmSak-pcDNA plasmid construct was used for the production of different mutants using the QuickChange Site-Directed Mutagenesis Kit (Stratagene) (according to the procedure described in [23]). A dead kinase version of SmSak (SmSak^DK^) was obtained by replacing the D_160_FG_162_ sequence by a D_160_ *N*A_162_ inactive motif using the primer 5′-CAAAATTGCTGAC**AATGCA**TTGGCTACTAAAATTG-3′ and its reverse complement. Constitutively active (SmSakT_175_D) or inactive (SmSakT_175_A) mutants were generated using 5′-GGTGAAGATCATAAA**GAC**ATGTGTGGGACACC-3′ and 5′-GGTGAAGATCATAAA**GCA**ATGTGTGGGACACC-3′primers and their reverse complement, respectively (modified nucleotides are in bold and underlined). A double mutant (SmSakT_175_D^DK^) was obtained by replacing the DFG motif by a D*N*A sequence into the SmSakT_175_D construct.

Myc-tagged *SmSak* construct was obtained following the excision of the SmSak full-length sequence from SmSak pcDNA construct by *EcoRI/NotI* double digestion and its further insertion in frame in pGBKT7 plasmid (Clontech) using the same restriction sites.

SmPlk1/SmSak hybrid proteins were obtained by the bi-directional exchange of the N terminal (containing the kinase domain, Kin1 or Kin4) and C terminal (containing PBD1 or PBD4) parts of SmPlk1 and SmSak, generating Kin1-PBD4 and Kin4-PBD1 mutants. To facilitate SmPlk1 and SmSak domain swap, a *Not1* restriction site was introduced in each sequence behind their kinase domains (Kin1 and Kin4 respectively) at positions corresponding to the residues 322 in SmPlk1 and 342 in SmSak. Site-directed mutagenesis of SmPlk1-pcDNA [23] and SmSak-pcDNA plasmids was performed using respectively the *Not1-*containing sequences 5′-CCTATTACCAGTG**GCGGCCGC**GCCCTTGGTAATG-3′ and 5′-CCCTTACTTTTTG**GCGGCCGC**ACAACCCTTGTC-3′ and their reverse complement as primers. Mutated plasmids were then submitted to a digestion by *NotI* and each linearized plasmid as well as *NotI/NotI* excised fragments containing respectively PBD1 and PBD4 were purified, then submitted to a ligation in order to obtain Kin1-PBD4 and Kin4-PBD1 in-frame sequences.

### Protein Expression in *Xenopus* Oocytes

cRNA encoding wild-type or mutant Plk proteins was synthesised *in vitro* from pcDNA or pGBKT7 constructs (that contain a T7 promoter sequence) using the T7mMessage mMachine Kit (Ambion, USA) following their linearization by *PmeI* (pcDNA) or *HindIII* (pGBKT7) restriction enzymes. cRNA transcribed from 1 µg of linearized plasmid was precipitated by 2.5 M LiCl, washed in 70% ethanol, resuspended in 20 µl water, then quantified by spectrophotometry. Finally, 1 µg of cRNA was analysed on a denaturating agarose gel, stained by ethidium bromide, in order to confirm the correct size of cRNA and the absence of abortive transcripts. cRNA solutions (1 mg/ml) were microinjected in stage VI oocytes of *Xenopus laevis* according to the protocol previously described [51]. Each oocyte was injected with 60 nl of cRNA in the equatorial region and incubated at 19°C in ND96 medium (96 mM NaCl, 2 mM KCl, 1 mM MgCl_2_, 1.8 mM CaCl_2_, 5 mM HEPES pH 7.4 supplemented with 50 µg/ml streptomycin/penicillin, 225 µg ml^−1^ sodium pyruvate, 30 µg/ml trypsin inhibitor) for 18 h. Germinal vesicle breakdown (GVBD) was detected by the appearance of a white spot at the centre of the animal pole. Blocking of endogenous Plx1 activity was performed by injecting 5 ng (in 25 nl) of anti-*Xenopus* Plk antibodies (AZ34, Santa Cruz Biotechnology, Inc., USA) one hour before the injection of cRNA encoding schistosome Plks or before the addition of the natural inducer of GVBD, progesterone (PG, 2 µg/ml).

For experiments of activation and induction of speckled phenotype, *in vitro*-matured oocytes which have been previously treated with PG for 15 h (and which are comparable to unfertilized eggs at metaphase II) were transferred in ND96 medium previously diluted with 4.8 vol of water and added with 1 µM final concentration of calcium ionophore A23187 (Calbiochem). Egg surface morphology was monitored over time under a binocular microscope.

### Western Blot Analyses and Immunoprecipitation

Western blot analyses of total oocyte homogenates were performed as described previously [64]. Blots were incubated with anti-V5 antibodies (1/5 000, Invitrogen), anti-*Xenopus* Plx1 (1/10 000) or anti-*Xenopus* Cdc25 (1/2 000) antibodies (both sera were kindly given by A. Kumagai, California Institute of Technology). Horseradish peroxidase (HRP)-labelled secondary antibodies were detected using the enhanced chemoluminescence western blotting detection system (GE Healthcare).

Immunoprecipitation of V5-tagged SmPlk1 and Myc-tagged SmSak proteins expressed in oocytes was performed according to the procedure described previously [51]. Following 24 h of expression, oocytes were lysed in buffer (50 mM HEPES pH 7.4, 500 mM NaCl, 0.05% SDS, 0.5% Triton X100, 5 mM MgCl2, 1 mg/ml bovine serum albumin, 10 µg/ml leupeptin, 10 µg/ml aprotinin, 10 µg/ml soybean trypsin inhibitor, 10 µg/ml benz-amidine, 1 mM PMSF, 1 mM sodium vanadate) and centrifuged at 4°C for 15 min at 10,000 g. Supernatants were incubated with anti-V5 or anti-Myc (1/100; Invitrogen) antibodies overnight at 4°C. Protein A-Sepharose beads (5 mg, Amersham Biosciences) were added for 1 h at 4°C. Immune complexes were collected by centrifugation, rinsed three times, resuspended in Laemmli sample buffer, and subjected to a 10% SDS-PAGE. Immune complexes were analyzed by Western blotting using anti-V5 or anti-myc (1/50,000) antibodies and the advanced ECL detection system (Amersham Biosciences).

### Quantitative RT-PCR

Total RNA was isolated from the different stages of *S. mansoni* with TRIzol® reagent (Invitrogen) according to the manufacturer’s instructions. cDNAs were obtained by reverse transcription of total RNA using the Thermoscript RT-PCR System (Invitrogen) and used as templates in triplicate assays for PCR amplification using the SYBR® Green PCR Master Mix and ABI PRISM 7000 sequence detection system (Applied Biosystems) as described previously [52]. Specific primers for SmSak (nucleotide (nt) 550–568; 631–651) and SmPlk1 (nt 310–330; 389–410) were designed by the Primer Express Program (Applied Biosystems). Primers specific for *S. mansoni* α-tubulin (GenBank No. M80214, nt 851–873; 904–925) were used as internal controls. For graphical representation of qPCR data, ΔCt (cycle threshold) values (ie Ct SmSak- Ct Tubulin) were calculated for each parasite stage, and the ΔCt value obtained for male worms was deducted from each value using the delta-delta Ct (ΔΔCt) method [65]. The ratio of SmSak/SmPlk1 transcripts was expressed for a given parasite stage by the value of 2^−ΔCt^, where ΔCt corresponds to the difference between the Ct value found for SmSak and that found for SmPlk1 in a given stage.

### *In situ* Hybridization

Adult worm pair sections were prepared and analyzed as previously described [66]. For hybridization, *in vitro* sense and antisense probes of SmSak (nt 2122–2594) were labeled with digoxigenin following the manufacturer instructions (Roche). Their size was controlled by gel electrophoresis and digoxigenin incorporation was confirmed on blots using alkaline phosphatase-conjugated anti-digoxigenin antibodies (Roche), naphthol-AS-phosphate, and Fast Red TR (Sigma). *In situ* hybridizations were performed for 16 h at 42°C. Parasite sections were stringently washed up to 0.5×SSC (saline salt citrate) and detection of digoxigenin-labelled transcripts was achieved as described for blots.

## Supporting Information

Figure S1 **Phylogenetic analysis of S** ***chistosoma mansoni*** **polo-like kinases.** *A.* Neighbour joining tree was generated using MEGA5 under Dayhoff model with 1000 bootstrap repetitions. Bootstrap values are indicated at the nodes. The scale bar corresponds to 0.2 substitution per site. Protein sequences of polo-like kinases of *Saccharomyces cerevisiae, Schizosaccharomyces pombe, Caenorhabditis elegans, Drosophila melanogaster, S. mansoni, Xenopus laevis, Mus musculus and Homo sapiens* were analyzed. *B.* Protein names and accession numbers are given.
(PDF)

Figure S2 **Protein sequence alignment of SmSak with other Sak/Plk4 kinases.** The SmSak sequence was aligned with that of human hPlk4 (Genbank accession No NP_055079.3), mouse mSak (AAC37648.1), Plx4 of *X. laevis* (NP_001083146.1) and DmSak (AAF51737.1) of *D. melanogaster* using ClustalW (DNASTAR Inc., Madison, WI, USA). Shaded areas correspond to residues which are identical (in black) or similar (in grey) in at least 75% of the aligned sequences.The serine/threonine kinase domain (boxed in red) contains the residues required for kinase activity GxGxFA, HRDLxLxN, DFG and GTPNYIxPE (red letters). The threonine residue within the T-loop (T_175_ in SmSak), whose phosphorylation induces kinase activation, is pointed out by a blue triangle. The cryptic polo-box (cry-pb) and polo-box (PB) are boxed respectively in dark and light blue. Amino acids involved in PB dimerization are indicated with red stars. Finally, the degron motif responsible for protein degradation [19] and located just behind the kinase domain is underlined in black.
(PDF)
