# Supplementary material for: SmSak, the Second Polo-Like Kinase of the Helminth Parasite Schistosoma mansoni: Conserved and Unexpected Roles in Meiosis
Source: PLoS One. 2012 Jun 29;7(6):e40045. doi: 10.1371/journal.pone.0040045 (PMC3386946; doi:10.1371/journal.pone.0040045)
Supplement: Figure S1 — Phylogenetic analysis of S chistosoma mansoni polo-like kinases. A. Neighbour joining tree was generated using MEGA5 under Dayhoff model with 1000 bootstrap repetitions. Bootstrap values are indicated at the nodes. The scale bar corresponds to 0.2 substitution per site. Protein sequences of polo-like kinases of Saccharomyces cerevisiae, Schizosaccharomyces pombe, Caenorhabditis elegans, Drosophila melanogaster, S. mansoni, Xenopus laevis, Mus musculus and Homo sapiens were analyzed. B. Protein names and accession numbers are given. (PDF) [file pone.0040045.s001.pdf]

A

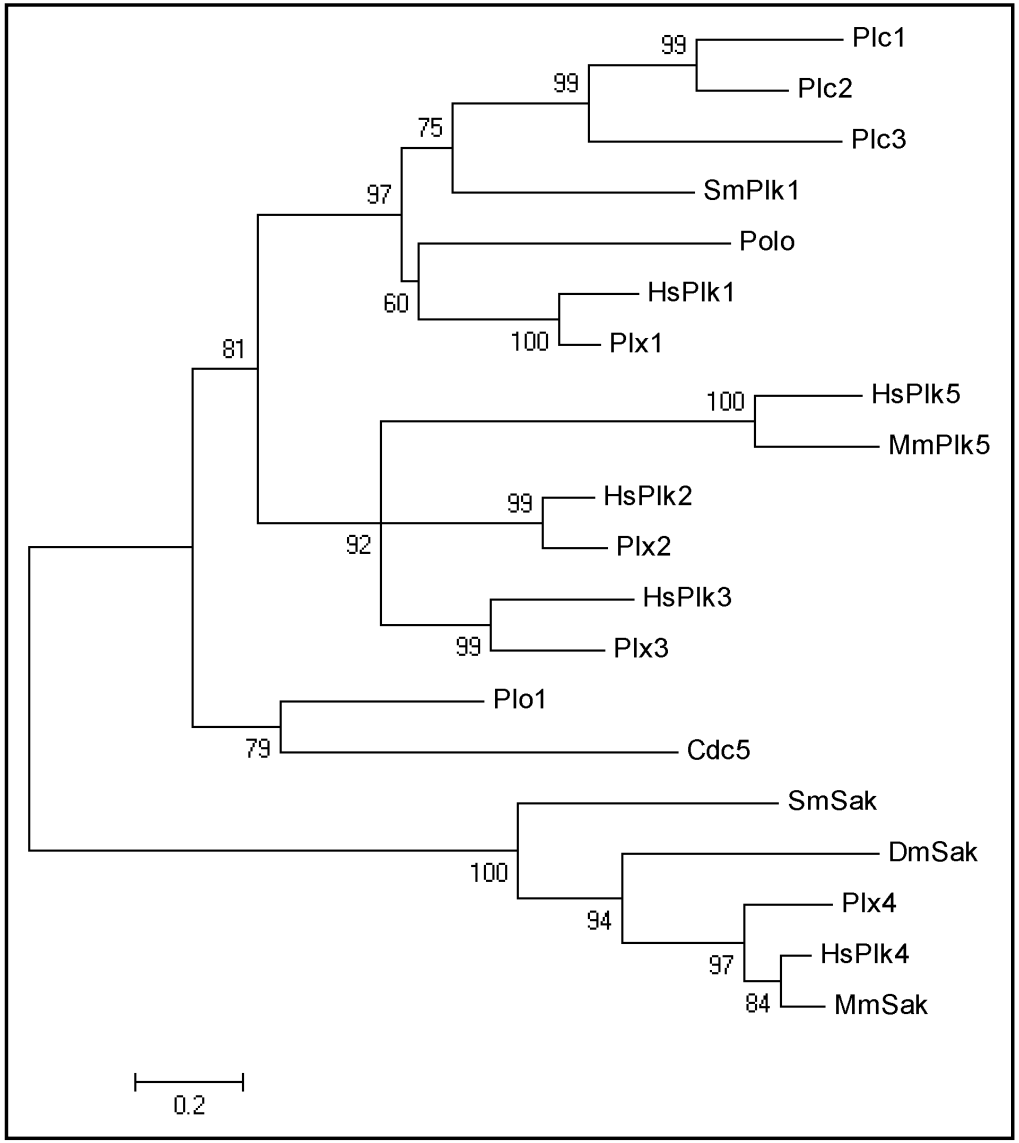

B

	Organisms	Polo-like kinases	GenBank Accession N°
Yeast	*S. pombe*	Plo1	CAB11167.1
	S. cerevisiae	Cdc5	P32562
Invertebrates	*C. elegans*	Plc1	P34331.3
		Plc2	Q9N2L7.1
		Plc3	Q20845.1
	D. melanogaster	DmPolo	P52304.2
		DmSak	AAF51737.1
	S. mansoni	SmPlk1	AAV49163.1
		SmSak	ADB12549.1
Vertebrates	*X. laevis*	Plx1	P70032.1
		Plx2	NP_001079435.1
		Plx3	AAL30176.1
		Plx4	NP_001083146.1
	M. musculus	MmSak	AAC37648.1
		MmPlk5	Q4FZD7.1
	H. sapiens	HsPlk1	NP_005021.2
		HsPlk2	NP_006613.2
		HsPlk3	NP_004064.2
		HsPlk4	NP_055079.3
		HsPlk5	NP_001230008.1
