# Supplementary material for: SmSak, the Second Polo-Like Kinase of the Helminth Parasite Schistosoma mansoni: Conserved and Unexpected Roles in Meiosis
Source: PLoS One. 2012 Jun 29;7(6):e40045. doi: 10.1371/journal.pone.0040045 (PMC3386946; doi:10.1371/journal.pone.0040045)
Supplement: Figure S2 — Protein sequence alignment of SmSak with other Sak/Plk4 kinases. The SmSak sequence was aligned with that of human hPlk4 (Genbank accession No NP_055079.3), mouse mSak (AAC37648.1), Plx4 of X. laevis (NP_001083146.1) and DmSak (AAF51737.1) of D. melanogaster using ClustalW (DNASTAR Inc., Madison, WI, USA). Shaded areas correspond to residues which are identical (in black) or similar (in grey) in at least 75% of the aligned sequences.The serine/threonine kinase domain (boxed in red) contains the residues required for kinase activity GxGxFA, HRDLxLxN, DFG and GTPNYIxPE (red letters). The threonine residue within the T-loop (T175 in SmSak), whose phosphorylation induces kinase activation, is pointed out by a blue triangle. The cryptic polo-box (cry-pb) and polo-box (PB) are boxed respectively in dark and light blue. Amino acids involved in PB dimerization are indicated with red stars. Finally, the degron motif responsible for protein degradation [19] and located just behind the kinase domain is underlined in black. (PDF) [file pone.0040045.s002.pdf]

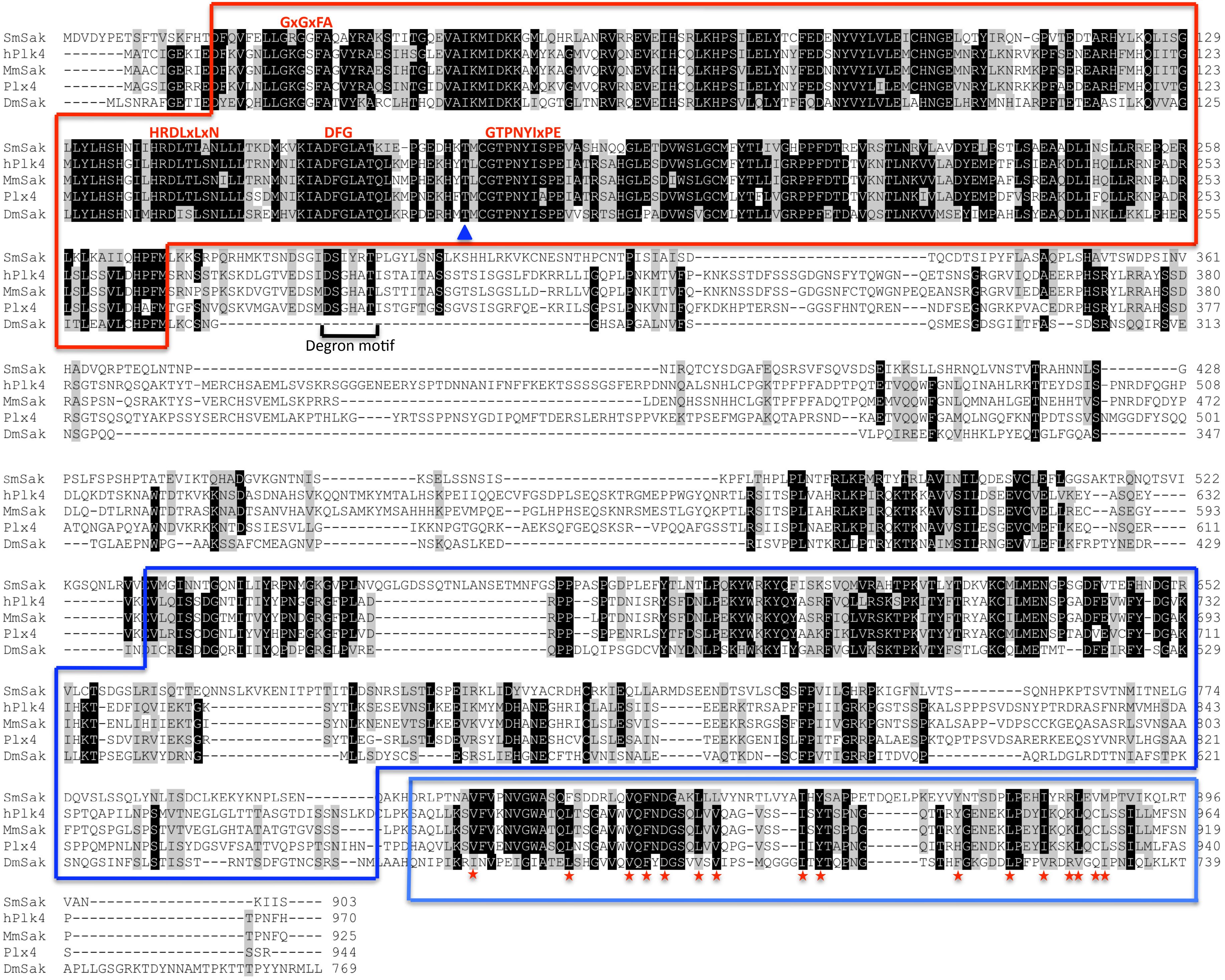
GxGxFA
SmSak MDVDYPETSFTVSKFHTDFQVFELLGRGGFAQAYRAKSTITGQEVAIKMIDKKGMLQHRLANRVRREVEIHSRLKHPSILELYTCFEDENYVYLVLEICHNGELQTYIRQN-GPVTEDTARHYLKQLISG 129
hPlk4 -------MATCIGEKIEDFKVGNLLGKGSFAGVYRAESIHTGLEVAIKMIDKKAMYKAGMVQRVQNEVKIHCQLKHPSILELYNYFEDSNYVYLVLEMCHNGEMNRYLKNRVKPFSENEARHFMHQIITG 123
MmSak -------MAACIGERIEDFKVGNLLGKGSFAGVYRAESIHTGLEVAIKMIDKKAMYKAGMVQRVQNEVKIHCQLKHPSVLELYNYFEDNNYVYLVLEMCHNGEMNRYLKNRMKPFSEREARHFMHQIITG 123
Plx4 -------MAGSIGERREDFKVLNLLGKGSFACVYRAQSINTGIDVAIKMIDKKAMQKVGMVQRVRNEVEIHCQLKHPSILELYNYFEDSNYVYLILEMCHNGEVNRYLKNRKKPFAEDEARHFMHQIVTG 123
DmSak -----MLSNRAFGETIEDYEVQHLLGKGGFATVYKARCLHTHQDVAIKMIDKKLIQGTGLTNRVRQEVEIHSRLKHPSVLQLYTFFQDANYVYLVLELAHNGELHRYMNHIARPFTETEAASILKQVVAG 125
HRDLxLxN
DFG
GTPNYIxPE
SmSak LLYLHSHNIIHRDLTLANLLLTKDMKVKIADFGLATKIE-PGEDHKTMCGTPNYISPEVASHNQQGLETDVWSLGCMFYTLIVGHPPFDTREVRSTLNRVLAVDYELPSTLSAEAADLINSLLRREPQER 258
hPlk4 MLYLHSHGILHRDLTLSNLLLTRNMNIKIADFGLATQLKMPHEKHYTLCGTPNYISPEIATRSAHGLESDVWSLGCMFYTLLIGRPPFDTDTVKNTLNKVVLADYEMPTFLSIEAKDLIHQLLRRNPADR 253
MmSak MLYLHSHGILHRDLTLSNILLTRNMNIKIADFGLATQLNMPHEKHYTLCGTPNYISPEIATRSAHGLESDIWSLGCMFYTLLIGRPPFDTDTVKNTLNKVVLADYEMPAFLSREAQDLIHQLLRRNPADR 253
Plx4 MLYLHSHGILHRDLTLSNLLLSSDMNIKIADFGLATQLKMPNEKHFTMCGTPNYIAPEIATRSAHGLESDVWSLGCMLYTFLVGRPPFDTDTVKNTLNKIVLADYEMPDFVSREAKDLIFQLLRKNPADR 253
DmSak LLYLHSHNIMHRDISLSNLLLSREMHVKIADFGLATQLKRPDERHMTMCGTPNYISPEVVSRTSHGLPADVWSVGCMLYTLLVGRPPFETDAVQSTLNKVVMSEYIMPAHLSYEAQDLINKLLKKLPHER 255
SmSak LKLKAIIQHPFMLKKSRPQRHMKTSNDSGIDSIYRTPLGYLSNSLKSHHLRKVKCNESNTHPCNTPISIAISD--------------------------TQCDTSIPYFLASAQPLSHAVTSWDPSINV 361
hPlk4 LSLSSVLDHPFMSRNSSTKSKDLGTVEDSIDSGHATISTAITASSSTSISGSLFDKRRLLIGQPLPNKMTVFP-KNKSSTDFSSSGDGNSFYTQWGN--QETSNSGRGRVIQDAEERPHSRYLRRAYSSD 380
MmSak LSLSSVLDHPFMSRNPSPKSKDVGTVEDSMDSGHATLSTTITASSGTSLSGSLLD-RRLLVGQPLPNKITVFQ-KNKNSSDFSS-GDGSNFCTQWGNPEQEANSRGRGRVIEDAEERPHSRYLRRAYSSD 380
Plx4 LSLSSVLDHAFMTGFSNVQSKVMGAVEDSMDSGHATISTGFTGSSGVSISGRFQE-KRILSGPSLPNKVNIFQFKDKHPTERSN--GGSFHNTQREN---NDFSEGNGRKPVACEDRPHSRYLRRAHSSD 377
DmSak ITLEAVLCHPFMLKCSNG---GHSAPGALNVES------------------------------QSMESGDSGIITFAS--SDSRNSQQIRSVE 313
Degron motif
SmSak HADVQRPTEQLNTNP---NIRQTCYSDGAFEQSRSVFSQVSDSEIKKSLLSHRNQLVNSTVTRAHNNLS---------G 428
hPlk4 RSGTSNRQSQAKTYT-MERCHSAEMLSVSKRSGGGENEERYSPTDNNANIFNFFKEKTSSSSGSFERPDNNQALSNHLCPGKTPFPFADPTPQTETVQQWFGNLQINAHLRKTTEYDSIS-PNRDFQGHP 508
MmSak RASPSN-QSRAKTYS-VERCHSVEMLSKPRRS---------------------------------------LDENQHSSNHHCLGKTPFPFADQTPQMEMVQQWFGNLQMNAHLGETNEHHTVS-PNRDFQDYP 472
Plx4 RSGTSQSQTYAKPSSYSERCHSVEMLAKPTHLKG----YRTSSPPNSYGDIPQMFTDERSLERHTSPPVKEKTPSEFMGPAKQTAPRSND--KAETVQQWFGAMQLNGQFKNTPDTSSVSNMGGDFYSQQ 501
DmSak NSGPQQ---VLPQIREEFKQVHHKLPYEQTGLFGQAS---------- 347
SmSak PSLFSPSHPTATEVIKTQHADGVKGNTNIS-----------KSELSSNSIS-------------------------KPFLTHPLPLNTFRLKPMRTYTRLAVINILQDESVCLEFLGGSAKTRQNQTSVI 522
hPlk4 DLQKDTSKNAWTDTKVKKNSDASDNAHSVKQQNTMKYMTALHSKPEIIQQECVFGSDPLSEQSKTRGMEPPWGYQNRTLRSITSPLVAHRLKPIRQKTKKAVVSILDSEEVCVELVKEY--ASQEY---- 632
MmSak DLQ-DTLRNAWTDTRASKNADTSANVHAVKQLSAMKYMSAHHHKPEVMPQE--PGLHPHSEQSKNRSMESTLGYQKPTLRSITSPLIAHRLKPIRQKTKKAVVSILDSEEVCVELLREC--ASEGY---- 593
Plx4 ATQNGAPQYAWNDVKRKKNTDSSIESVLLG----------IKKNPGTGQRK--AEKSQFGEQSKSR--VPQQAFGSSTLRSIISPLNAERLKPIRQKTKNAVVSILESGEVCMEFLKEQ--NSQER---- 611
DmSak ---TGLAEPNWPG--AAKSSAFCMEAGNVP-----------NSKQASLKED----------------------------RISVPPLNTKRLLPTRYKTKNAIMSIIRNGEVVLEFLKFRPTYNEDR---- 429
SmSak KGSQNLRVVEVMGINNTGQNILIYRPNMGKGVPLNVQGLGDSSQTNLANSETMNFGSPPPASPGDPLEFYTLNTLPQKYWRKYQFISKSVQMVRAHTPKVTLYTDKVKCMLMENGPSGDFVTEFHNDGTR 652
hPlk4 -------VKEVLQISSDGNTITIYYPNGGRGFPLAD--------------------RPP--SPTDNISRYSFDNLPEKYWRKYQYASRFVQLLRSKSPKITYFTRYAKCILMENSPGADFEVWFY-DGVK 732
MmSak -------VKEVLQISSDGTMITVYYPNDGRGFPLAD--------------------RPP--LPTDNISRYSFDNLPEKYWRKYQYASRFIQLVRSKTPKITYFTRYAKCILMENSPGADFEVWFY-DGAK 693
Plx4 -------VKEVLRISCDGNLIYVYHPNEGKGFPLVD--------------------RPP--SPPENRLSYTFDSLPEKYWKKYQYAAKFIKLVRSKTPKVTYYTRYAKCMLMENSPTADVEVCFY-DGAK 711
DmSak -------INDICRISDDGQRIIIYQPDPGRGLPVRE--------------------QPPDLQIPSGDCVYNYDNLPSKHWKKYIYGARFVGLVKSKTPKVTYFSTLGKCQLMETMT--DFEIRFY-SGAK 529
SmSak VLCTSDGSLRISQTTEQNNSLKVKENITPTTITLDSNRSLSTLSPEIRKLIDYVYACRDHCRKIEQLLARMDSEENDTSVLSCSSFPVILGHRPKIGFNLVTS--------SQNHPKPTSVTNMITNELG 774
hPlk4 IHKT-EDFIQVIEKTGK-------------SYTLKSESEVNSLKEEIKMYMDHANEGHRICLALESIIS-----EEERKTRSAPFFPIIIGRKPGSTSSPKALSPPPSVDSNYPTRDRASFNRMVMHSDA 843
MmSak IHKT-ENLIHIIEKTGI-------------SYNLKNENEVTSLKEEVKVYMDHANEGHRICLSLESVIS-----EEEKRSRGSSFFPIIVGRKPGNTSSPKALSAPP-VDPSCCKGEQASASRLSVNSAA 803
Plx4 IHKT-SDVIRVIEKSGR-------------SYTLEG-SRLSTLSDEVRSYLDHANESHCVCLSLESAIN-----TEEKKGENISLFPITFGRRPALAESPKTQPTPSVDSARERKEEQSYVNRVLHGSAA 821
DmSak LLKTPSEGLKVYDRNG---------------MLLSDYSCS----ESRSLIEHGNECFTHCVNISNALE-----VAQTKDN--SCFPVTIGRRPITDVQP-----------AQRLDGLRDTTNIAFSTPK 621
SmSak DQVSLSSQLYNLISDCLKEKYKNPLSEN--------QAKHDRLPTNAVFVPNVGWASQFSDDRLQVQFNDGAKLLLVYNRTLVYAIHYSAPPETDQELPKEYVYNTSDPLPEHIYRRLEVMPTVIKQLRT 896
hPlk4 SPTQAPILNPSMVTNEGLGLTTTASGTDISSNSLKDCLPKSAQLLKSVFVKNVGWATQLTSGAVWVQFNDGSQLVVQAG-VSS--ISYTSPNG------QTTRYGENEKLPDYIKQKLQCLSSILLMFSN 964
MmSak FPTQSPGLSPSTVTVEGLGHTATATGTGVSSS-----LPKSAQLLKSVFVKNVGWATQLTSGAVWVQFNDGSQLVVQAG-VSS--ISYTSPDG------QTTRYGENEKLPEYIKQKLQCLSSILLMFSN 919
Plx4 SPPQMPNLNPSLISYDGSVFSATTVQPSPTSNIHN--TPDHAQVLKSVFVENVGWASQLNSGAVWVQFNDGSQLVVQPG-VSS--IIYTAPNG------QITRHGENDKLPEYIKSKLQCLSSILMLFAS 940
DmSak SNQGSINFSLSTISST---RNTSDFGTNCSRS--NMLAAHQNIPIKRINVPEIGIATELSHGVVQVQFYDGSVVSVIPS-MQGGGITYTQPNG------TSTHFGKGDDLPFPVRDRVGQIPNIQLKLKT 739
SmSak VAN-------------------KIIS---- 903
hPlk4 P--------------------TPNFH---- 970
MmSak P--------------------TPNFQ---- 925
Plx4 S--------------------SSR------ 944
DmSak APLLGSGRKTDYNNAMTPKTTTPYYNRMLL 769
